# The safety and efficacy of fibrin sealant for thyroidectomy: a systematic review and meta-analysis of randomized controlled trials

**DOI:** 10.3389/fsurg.2023.1149882

**Published:** 2023-06-20

**Authors:** Heru Sutanto Koerniawan, Valeska Siulinda Candrawinata, Teddy Tjahyanto, Nicholas Jason Wijaya, Aulia Wiratama Putra, Jeremiah Hilkiah Wijaya

**Affiliations:** ^1^ Department of Surgery, Universitas Pelita Harapan, Tangerang, Indonesia; ^2^ Department of Medicine, Universitas Tarumanagara, Jakarta, Indonesia; ^3^ Department of Medicine, Universitas Indonesia, Jakarta, Indonesia; ^4^ Department of Medicine, Universitas Trisakti, Jakarta, Indonesia

**Keywords:** fibrin sealant, thyroidectomy, randomized controlled trials, volume drainage, systematic review

## Abstract

Fibrin sealants have recently been thoroughly studied in several surgical specialties; however, results are conflicting. We aimed to examine the safety and efficacy of fibrin sealant patients having thyroidectomies. A thorough, systematic literature search was carried out using the terms thyroidectomy and fibrin sealant using PubMed, Cochrane Library, and Clinicaltrials.gov on December 25, 2022. The primary outcome of interest in this review was the amount of drainage, whereas hospitalization, the length of drain retention, and temporary dysphonia were secondary outcomes. Our meta-analysis (*n* = 249) showed that application of fibrin sealant is associated with lesser total drainage [SMD −2.76 (−4.83, −0.69); *P* = 0.009; I2 97%], but not with retention time of drainage [SMD −2.35 (−4.71, 0.01); *P* = 0.05; I2 98%], hospitalization time [SMD −1.65 (−3.70, 0.41); *P* = 0.12; I2 97%], and transient dysphonia [RR 1.01 (0.27, 3.82); *P* = 0.99; I2 0%]. The systematic review found that the use of fibrin sealant in thyroid surgery is positive in total volume drainage but not with the retention time of drainage, hospitalization time, and transient dysphonia. It is notable to remember that this interpretation is complicated by uneven, occasionally subpar technique and trial reporting, according to this systematic review's findings.

## 1. Introduction

Thrombin and fibrinogen make up the two components of fibrin sealant (1). The final stable form of the substance, insoluble fibrin, is produced by the conversion of fibrinogen by thrombin in the presence of minute amounts of calcium and factor XIII. These products are used to seal biological tissues as two-component (i.e., fibrinogen and thrombin) liquid glue or as a two-component dry patch. Liquid glue formulations include Tisseel or Tissucol, Evicel, and dry patch products such as Tachosil and Tachocomb (2, 3). One of the earliest fibrin sealants among these products, Tisseel/Tissucol has been available for more than 30 years—it was originally offered on the European market in 1978 (4). There are two main active component groups in Tisseel. The first is the human thrombin and calcium chloride; the second is the human thrombin, synthetic aprotinin (anti-fibrinolytic), and Factor XIII (5, 6). When the two are combined and applied to tissue, the fibrinogen and thrombin create a fibrin clot that clings to the tissue, stops bleeding, and serves as bio glue.

Even though bleeding following thyroid surgery is uncommon, it can be fatal and necessitate an immediate reoperation. This fear makes surgeons do routine drains following any thyroid surgery (7). Although the presence of vascularized remnant tissue in partial thyroidectomy or Graves’ disease may increase the risk of bleeding, postoperative bleeding has been documented to occur as infrequently as 0.3% to 1% after thyroidectomy (8, 9).

The potential clinical benefits of fibrin sealant to patients and healthcare organizations are significant, which include a decrease in the frequency of postoperative complications (such as the formation of haematomas or seromas and infections) and a decrease in surgical wound drainage, which can reduce or eliminate the need for surgical drains (1). Woods et al. showed in a comprehensive review and meta-analysis of wound drain used following thyroid surgery that they were not always necessary and increased infection rates, postoperative pain, and length of hospital stay (10). On the other hand, there is a worry that some forms of fibrin sealants may have negative consequences, such as leaving deadly air pockets inside the body. Warnings have been issued by the Food and Drug Administration (FDA) in the USA regarding the development of potentially fatal air or gas emboli following the use of fibrin sealant aerosol sprays during surgery (11). The risks of employing sprays too closely to exposed tissue surfaces and at pressures higher than those advised by the makers have been made clear to users of the goods. Nevertheless, there is a lack of information from RCTs about damages despite safety concerns. In a multicenter RCT carried out in three institutions in Italy, the rate of unfavorable outcomes was compared in a group of patients receiving fibrin sealants as an adjuvant for preventing air leaks in patients having lung resection (12). Following these surgeries, broncopleural fistulas and air leaks are also frequent side effects (13, 14). The researchers discovered that there was no statistically significant difference in the rate of adverse events between patients who received fibrin sealant and those who did not, with a follow-up duration of 30—40 days (12).

Three published systematic studies have mainly addressed tonsillectomy and rhytidectomy when discussing tissue adhesives, not always fibrin sealant, in soft-tissue surgery of the head and neck (15–17). The most current systematic analysis on the use of tissue adhesives in rhytidectomy revealed that their usage considerably decreased the rate of haematoma formation and decreased the volume of surgical drainage (16). Results, however, have needed to be more consistent and frequently unfairly impacted by poor study design. To address these concerns, we conducted this systematic review and meta-analysis. We aimed to examine the safety and efficacy of fibrin sealant in patients undergoing thyroidectomies.

## 2. Materials and methods

This systematic review and meta-analysis adhered to Preferred Reporting Items for Systematic Reviews and Meta-Analyses (PRISMA) and was conducted manually (18). However, we did not register our study to the PROSPERO. A thorough, systematic literature search was carried out using the terms thyroidectomy and fibrin sealant using PubMed, Cochrane Library, and Clinicaltrials.gov. Table 1 displays specific search queries from each database. All authors did the literature search on December 25, 2022. Two authors (H.S. and V.S.) read the titles and abstracts and evaluated the possibly eligible papers using the inclusion and exclusion criteria. We settled any disagreements that arose during this procedure.

**Table 1:** Search strategy performed in each electronic database.

Electronic database	Search terms
PubMed	(“fibrin tissue adhesive"[MeSH Terms] OR (“fibrin"[All Fields] AND “tissue"[All Fields] AND “adhesive"[All Fields]) OR “fibrin tissue adhesive"[All Fields] OR (“fibrin"[All Fields] AND “sealant"[All Fields]) OR “fibrin sealant"[All Fields]) AND (((“thyroid gland"[MeSH Terms] OR (“thyroid"[All Fields] AND “gland"[All Fields]) OR “thyroid gland"[All Fields] OR “thyroid"[All Fields] OR “thyroid usp"[MeSH Terms] OR (“thyroid"[All Fields] AND “usp"[All Fields]) OR “thyroid usp"[All Fields] OR “thyroids"[All Fields] OR “thyroid s"[All Fields] OR “thyroidal"[All Fields] OR “thyroideal"[All Fields] OR “thyroidism"[All Fields] OR “thyroiditis"[MeSH Terms] OR “thyroiditis"[All Fields] OR “thyroiditides"[All Fields]) AND (“surgery"[MeSH Subheading] OR “surgery"[All Fields] OR “surgical procedures, operative"[MeSH Terms] OR (“surgical"[All Fields] AND “procedures"[All Fields] AND “operative"[All Fields]) OR “operative surgical procedures"[All Fields] OR “general surgery"[MeSH Terms] OR (“general"[All Fields] AND “surgery"[All Fields]) OR “general surgery"[All Fields] OR “surgery s"[All Fields] OR “surgerys"[All Fields] OR “surgeries"[All Fields])) OR (“thyroidectomy"[MeSH Terms] OR “thyroidectomy"[All Fields] OR “thyroidectomies"[All Fields]) OR (“hemithyroidectomies"[All Fields] OR “hemithyroidectomy"[All Fields]))
Cochrane Library	Fibrin sealant AND thyroid surgery OR thyroidectomy OR hemithyroidectomy
ClinicalTrials.gov	Fibrin sealant AND thyroid surgery OR thyroidectomy OR hemithyroidectomy

We included any published randomized clinical trials (RCTs) comparing fibrin sealant to control group, published in any year, written in any language, and having adult patients of any gender or ethnicity undergoing thyroid surgery that would typically necessitate the insertion of a surgical drain. RCTs with patients with fibrin sealant were used to cover the surgical dead space to reach underlying structures. Studies that were conference papers or abstracts-only publications, non-research letters, reviews, and editorial or commentaries were excluded.

The primary outcome of interest in this review was the amount of drainage, whereas hospitalization, the length of drain retention, and temporary dysphonia were secondary outcomes. We extracted the following details from each eligible study: first author, study design, fibrin sealant information, sample size, type of thyroid surgery, age, sex, and complications rate. We used a spreadsheet with predetermined column titles for each data entry to gather data. Formal requests were made to the principal investigators of included studies in cases where crucial information was missing.

We assessed the risk of bias independently, adhering to the Cochrane Risk of Bias (RoB) Assessment version 2 for RCTs. Any discrepancies were resolved by discussion.

We employed the inverse variance approach to evaluate continuous variables, and the pooled effect estimate was presented as standardized mean differences (SMD) with its standard deviation (SD). The risk ratios (RRs) and 95% confidence intervals (CIs) for dichotomous variables were calculated using the Mantel-Haenszel algorithm. In this meta-analysis, all *p* values were two-tailed, and the statistical threshold for significance was set at 0.05 (regardless of the heterogeneity, which is set at 0.10). To determine the origin of heterogeneity, We conducted a leave-one-out sensitivity analysis. Inverted funnel-plot analysis was used to evaluate the risk of publication bias qualitatively.

## 3. Results

**Figure 1:**
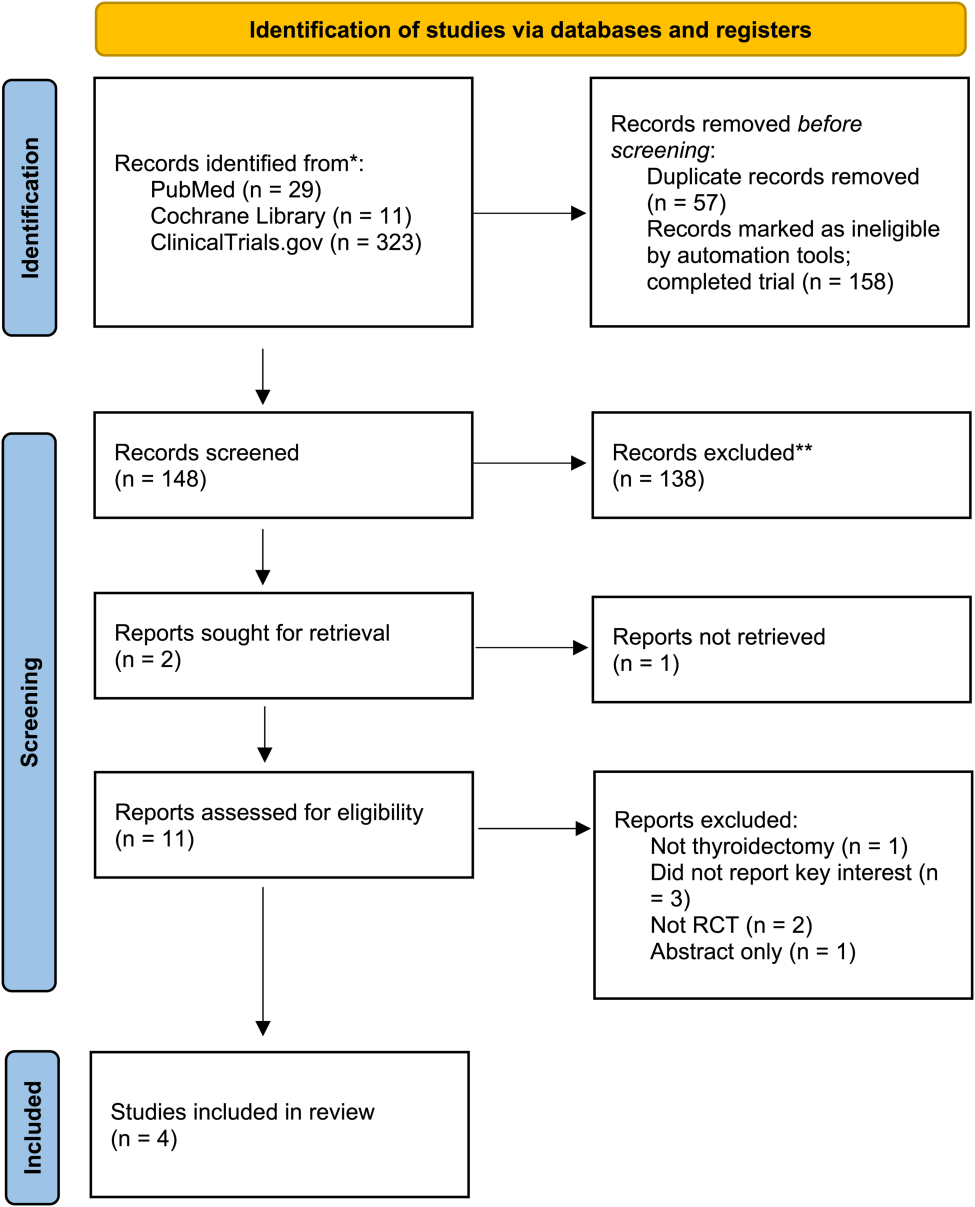
PRISMA flow diagram for this systematic review and meta-analysis.

After eliminating duplicate results, 206 records were left from the initial search's 363 records. We disregarded one hundred forty-eight records after title/abstract screening. We eliminated seven full-text articles after 11 full-text articles were assessed for eligibility due to the following reasons: did not report the key interests, not RCT, papers that reported other procedures, and an abstract published in a journal's supplementary file as it is presented in a conference. A total of 249 patients from 4 RCTs were included in this systematic review and meta-analysis (19–22). The most significant result was from Kim et al. (*n* = 78) showing the largest total drainage (93.5 ± 30.7 vs. 105.7 ± 31.2 ml for control and intervention group, respectively). However, the time to remove the drain was not shortened by the utilization of fibrin sealant (20). The PRISMA flow is depicted in Figure 1, and detailed characteristics of included studies are shown in Table 2.

**Table 2:** Summary characteristics of included studies (*n* = 249).

Study ID	Total cohort (*n*)	Total male (*n*)	Intervention details	Control details	Age (years)	Total thyroidectomy (*n*)	Total drainage (ml)	Funding
Vidal-Pérez 2016	30 vs. 30	5 vs. 9	Tissucol FS	No FS	52 ± 8 vs. 50 ± 7	30 vs. 30	51 (29–72) vs. 110 (74–132)	
Uwiera 2005	26 vs. 30	4 vs. 11	Tisseel FS	No FS	49 (27–73) vs. 50 (26–66)	16 vs. 9	53.4 ± 7.5 vs. 24.5 ± 4.6	
Kim 2012	38 vs. 40	6 vs. 13	Berplast P FS	No FS	47.8 ± 9.6 vs. 50.8 ± 10.8	38 vs. 40	93.5 ± 30.7 vs 105.7 ± 31.2	Samsung Medical Center
NCT01226914	28 vs. 27	4 vs. 3	Evicel FS	Saline Placebo	48.8 ± 15.2 vs. 51.9 ± 11.5	18 vs. 17	96.3 (73.3–139.3) vs 120 (68.8–161.5)	Ethicon, Inc.

**Figure 2:**
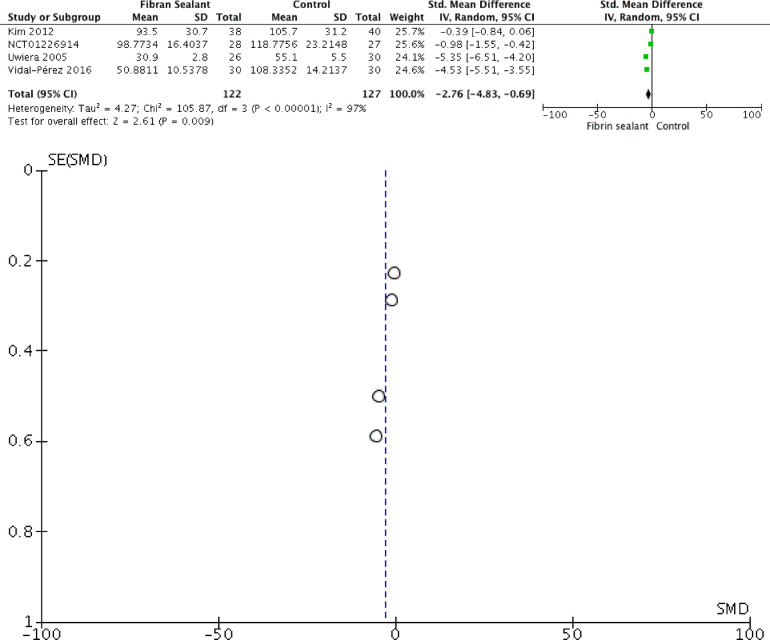
Forest and funnel plot for total drainage.

**Figure 3:**
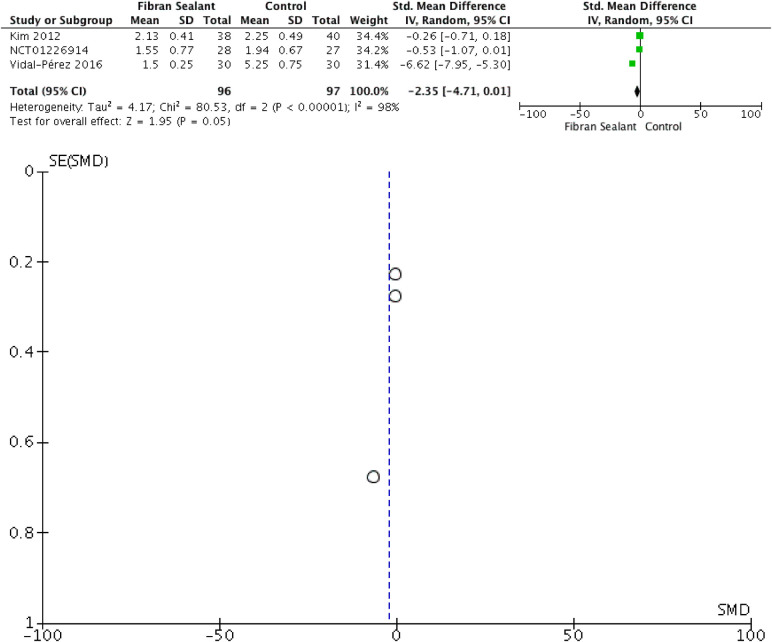
Meta-analysis for retention time of drainage between intervention and control arm.

**Figure 4:**
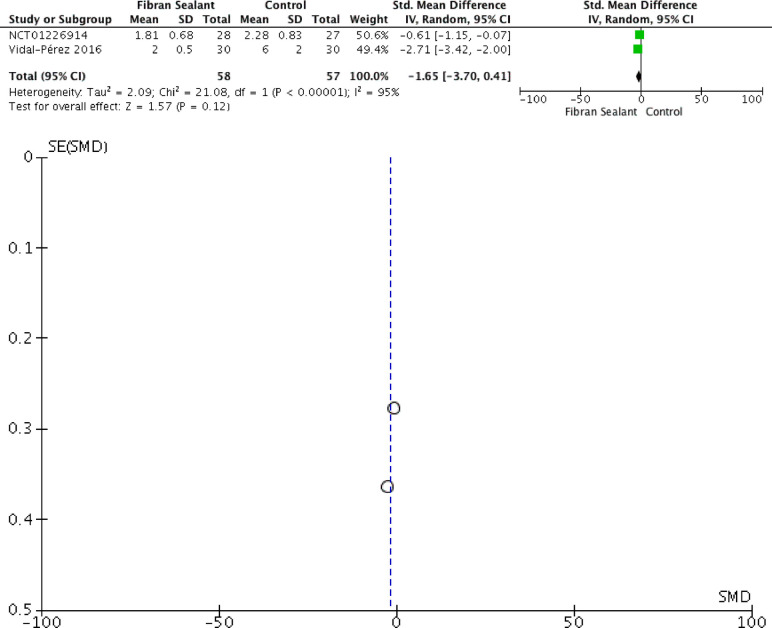
Meta-analysis for hospitalization time.

**Figure 5:**
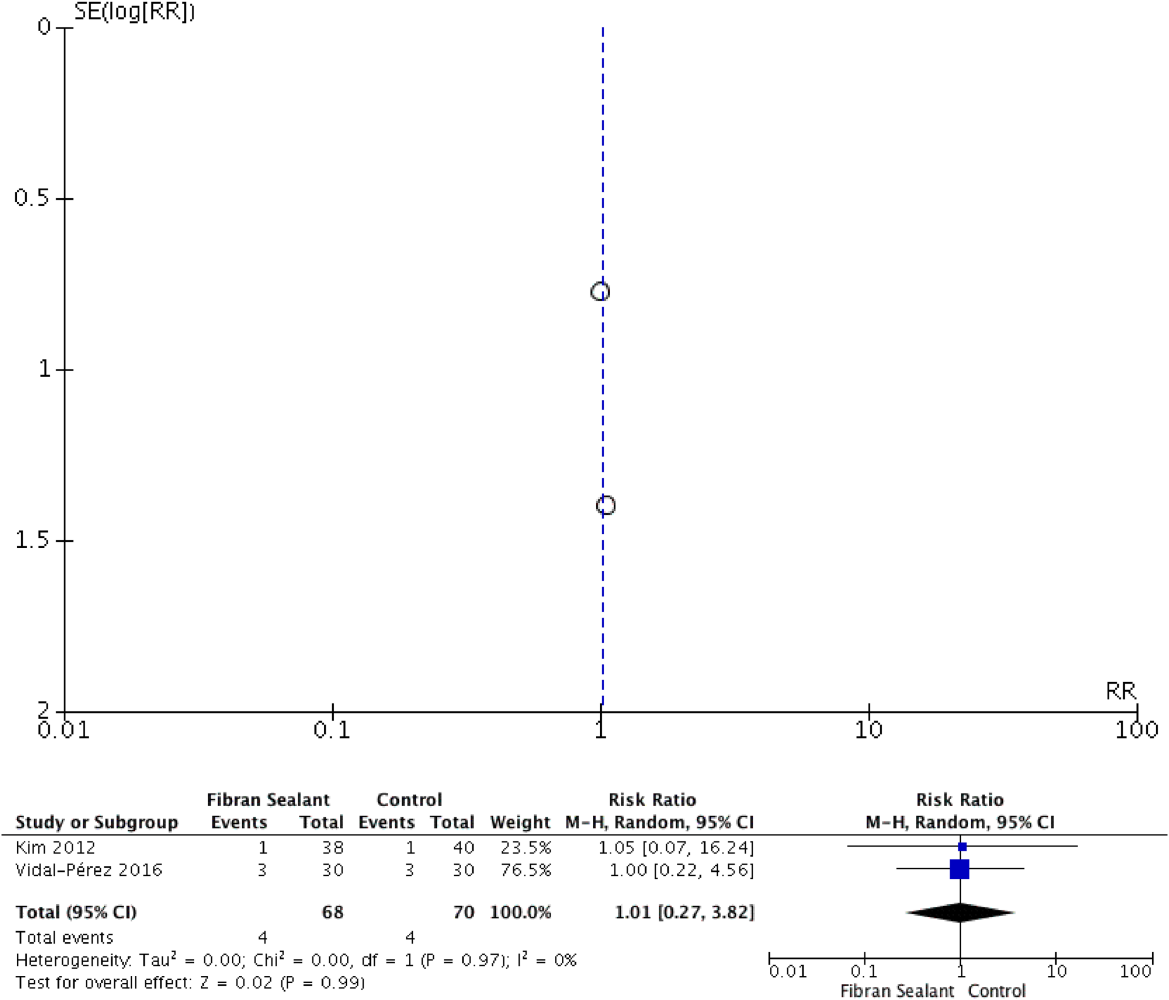
Forest plot and meta-analysis for patients experiencing transient dysphonia.

Our meta-analysis showed that application of fibrin sealant is associated with lesser total drainage [SMD −2.76 (−4.83, −0.69); *P* = 0.009; I2 97%], but not with retention time of drainage [SMD −2.35 (−4.71, 0.01); *P* = 0.05; I2 98%], hospitalization time [SMD −1.65 (−3.70, 0.41); *P* = 0.12; I2 97%], and transient dysphonia [RR 1.01 (0.27, 3.82); *P* = 0.99; I2 0%]. Meta-analyses can be seen in Figures 2–5 for total drainage, retention time of drainage, hospitalization time, and transient dysphonia, respectively.

### 3.1. Risk of bias

**Figure 6:**
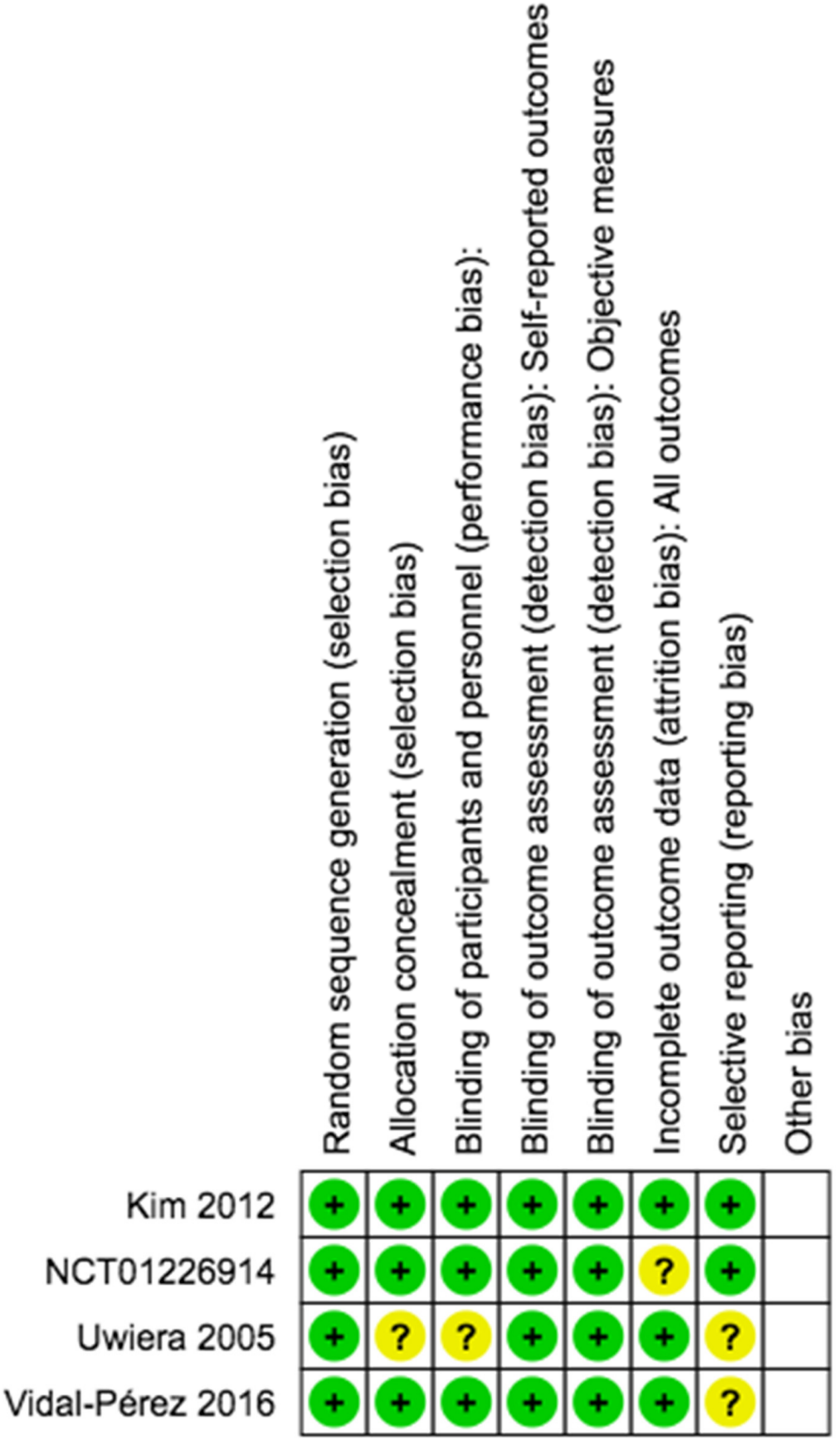
Risk of bias assessment version 2.

The relationship between fibrin sealant and thyroidectomy was visualized using a funnel-plot analysis as a qualitatively symmetrical inverted funnel-plot. Our Risk of Bias Cochrane version 2 also showed that all included RCTs were of low risk of bias. Detailed risk of bias were represented in Figure 6.

## 4. Discussions

Most surgical procedures have traditionally employed drains (23, 24). It has been a standard procedure to drain wounds following thyroid and parathyroid surgery. However, in neck surgeries, the likelihood of a significant postoperative haemorrhage is relatively low and does not appear to rise in the absence of drains (25, 26). The primary uses of a drain have historically been to prevent postoperative problems by removing lymphatic or postoperative bleeding and notifying the surgeon of any early postoperative bleeding. A surgeon typically inserts drains to stop bleeding and detect it early after thyroid surgeries. However, drain implantation increases the risk of infection and lengthens the hospital stay (27, 28). Patients are typically released from the hospital on the day of surgery because it is known that the risk of respiratory failure brought on by a haematoma is highest in the first six hours following thyroid surgery (29). Additionally, the drains may cause more drainage because of the inflammation they cause by simply being there (30, 31). The lymphatics may not be able to shut off due to the vacuum caused by the drain's negative pressure, which would increase seroma production and drainage. According to Schoretsanitis et al., the non-drained group's VAS score is reduced by about 50%. Most of the non-drained patients in this trial also saw a significant reduction in postoperative pain, particularly on postoperative day 1 (32). These findings demonstrated that drain placement might be directly related to the patient's postoperative discomfort by raising postoperative pain. The last concern is about the incidence of hematomas or seromas formation following thyroidectomy. Surgeons have become interested in having no drainage after thyroid surgery. However, the volume that drains spontaneously after surgery could not be predicted, therefore research to date have been constrained. An interesting study by Woo et al. from South Korea revealed that there was no increase in the likelihood of hematoma or seroma formation when a negative pressure drain was used to improve drainage during the first 24–48 h postoperatively (31).

Our study showed that applying fibrin sealant is associated with lesser overall drainage. A prospective RCT conducted by Docimo et al. assess the various hemostatic approaches in patients undergoing total thyroidectomy. In the intervention cohort receiving hemostatic agent was found to be effective at reducing postsurgical drain output and provides a complementary hemostatic approach in patients undergoing total thyroidectomy (48 ± 19.2 vs. 98.3 ± 23.1 ml in intervention and control group, respectively; 95% CI −63.2 to −31.2; *p*-value < 0.0001) (33). While other outcomes of interest, which include retention time of drainage, hospitalization time, and transient dysphonia, did not result in a statistically significant manner. Previous research revealed that the first 6 h following thyroid surgery have the highest risk of respiratory failure due to hemorrhage, which makes it discouraged to discharge patients on the day of surgery (34, 35). The length of hospitalization is a key factor in cost analysis, and numerous studies have demonstrated the connection between the length of hospitalization and the use of drains (18, 35, 36). In contrast to their potential to shorten hospital stays and increase discomfort, drains have not demonstrated the ability to reduce postoperative complications.

Regarding mean total drainage volume, there was significant statistical heterogeneity in the sub-group analysis of thyroidectomy trials. The different cut-off volumes may have caused this heterogeneity for drain removal used in the studies by Hornig et al. and Uwiera et al. (10 ml/8 hr and 10 ml/24 hr, respectively) (19, 21). The considerable statistical heterogeneity tempers the significant reduction of 36.36 ml in the mean total drainage volume for thyroidectomy in the fibrin sealant arm compared to the control. In contrast to a meta-analysis which showed that not using drains was safe and may even be beneficial, Hornig et al. and Uwiera et al. reported a mean total drainage volume ranging from about 70 — 120 ml (21). One would expect 70 — 120 ml in the anterior neck to be clinically apparent and require aspiration or evacuation. It is unknown whether this discrepancy results from the wound's closed suction drain's stimulating impact. It is challenging to make a case for employing fibrin sealant because Woods et al. have demonstrated that drains are not typically necessary in thyroid surgery (10). According to the current meta-analysis, fibrin sealant may reduce drainage volume, but this has yet to result in a noticeably different clinical outcome (both in the pooled and individual study analysis) (37). Other than stating that fibrin sealant is safe to employ, the study's findings do little to alter the practices of surgeons who already do drainless surgery. The use of fibrin sealant in patients more susceptible to problems needs more research.

In thyroid and parathyroid surgery, fibrin sealants offer a comparative advantage over under-flap suction. The agony patients experience when a drain is removed is also avoided using fibrin glue, which is also less expensive. According to Patel et al., the use of fibrin glue led to a statistically significant reduction in the number of time patients needed to spend in the hospital after undergoing both forms of surgery (124 patients who had undergone thyroidectomy and 47 patients who had undergone parathyroidectomy; *p* = 0.022 and 0.033, respectively). Another crucial point is that a pyriform sinus fistula-caused instance of recurrent suppurative thyroiditis was successfully treated by injecting fibrin glue (38).

### 4.1. Adverse reactions

Adverse events suspected to be connected to fibrin sealants were reported in several RCTs in different surgical specialities. Death following the use of fibrin sealant may be connected to the use of fibrin sealant in upper GI tract surgery caused by a significant bleed; however, there was no evidence of bleeding at the intended bleeding site. Various modest adverse events, such as mild cellulitis and mild seroma, anaemia, urine extravasation, incision site problems, and a slight generalized skin rash, were also mentioned in other RCTs. Excessive discomfort, scar pain, testicular pain hydrocele, and post-procedural hemorrhage antibodies all had varying degrees of severity. The severity of adverse events or their relationship to the use of fibrin sealant was not disclosed. After surgery, unfavourable outcomes happened instantly, after two hours, after 24 h, after 14 days, and 32 days (39).

### 4.2. Clinical implication

Human fibrinogen and its various constituent parts create fibrin glue, a sticky biological substance (40). By boosting homeostasis and angiogenesis and inducing macrophages, which play a role in fibroblast proliferation and collagen formation at the wound site, fibrin glue aids in wound healing (41, 42). In thyroid and parathyroid surgery, fibrin sealants offer a comparative advantage over under-flap suction (43–45). The agony patients experience when a drain is removed is also avoided using fibrin glue, which is also less expensive.

### 4.3. Limitation

The publication bias shown by the funnel plot represents a limitation of this systematic review and meta-analysis due to the small number of studies included. Due to the novelty and dearth of RCTs using fibrin sealant, the sample size was similarly modest. Although fibrin sealant tended to shorten hospital stays, this was not statistically significant and was confounded by solid statistical heterogeneity. It could not do a meta-analysis of postoperative pain because none of the included studies covered it. Lastly, we were not able to conduct a cost-benefit analysis.

In conclusion, the systematic review found that the use of fibrin sealant in thyroid surgery is positive in total volume drainage but not with the retention time of drainage, hospitalization time, and transient dysphonia. It is notable to remember that this interpretation is complicated by uneven, occasionally subpar technique and trial reporting, according to this systematic review's findings. As a result, it is inescapably difficult to draw definitive conclusions from meta-analyses. Additional clinical trials using sound methodology are required. This is especially true for lateral neck dissection, where there are few randomized data and where the potential for the most significant benefit is there.
